# Advancing small-angle X-ray scattering for complex metallic systems: Ti_2_Cu precipitation in a martensitic near-α Ti alloy

**DOI:** 10.1107/S1600576725008489

**Published:** 2025-11-04

**Authors:** David Obersteiner, Sabine C. Bodner, Helmut Clemens, Andreas Landefeld, Ehsan Farabi, Sophie Primig, Peter Staron, José L. Neves, Thomas Klein, Michael Musi

**Affiliations:** a Department of Materials Science, Chair of Physical Metallurgy, Montanuniversität Leoben, Franz Josef-Straße 18, 8700 Leoben, Austria; b School of Materials Science and Engineering UNSW Sydney Kensington 2052 NSW Australia; c Institute of Materials Physics, Helmholtz-Zentrum Hereon, Max-Planck Straße 1, 21502 Geesthacht, Germany; d LKR Light Metals Technologies AIT Austrian Institute of Technology Lamprechtshausenerstraße 61 5282 Braunau am Inn-Ranshofen Austria; Argonne National Laboratory, USA

**Keywords:** small-angle X-ray scattering, titanium alloys, precipitation kinetics, martensitic microstructure, methodology

## Abstract

Small-angle X-ray scattering (SAXS) experiments performed at synchrotron radiation sources enable the *in situ* study of precipitation behaviour, providing crucial information for alloy design. Numerous studies have demonstrated the successful use of SAXS to investigate precipitation phenomena across a wide range of complex metallic systems. Nevertheless, despite its advantages, the application of SAXS remains often overlooked owing to the challenges associated with data analysis, especially under non-isothermal conditions in which a metal matrix is continuously evolving due to simultaneous phase transformations and microstructural coarsening. This work presents a novel SAXS modelling approach developed for the quantitative evaluation of precipitate formation in metallic systems exhibiting scattering signals superimposed on directionally streaked signals originating from the embedding matrix, as commonly found in martensitic microstructures. Two-dimensional synchrotron data recorded during a continuous *in situ* heating experiment on a Cu- and Si-containing near-α Ti alloy are used to demonstrate how the evolving SAXS signal can be separated into matrix- and precipitation-related contributions. A Guinier–Porod function linked to a grain coarsening model was used to describe the background signal from the matrix, while Ti_2_Cu precipitates were modelled using an ellipsoidal model function combined with a lognormal size distribution. This combined approach enabled the evaluation of precipitate volume fraction and size distribution throughout the heat treatment. The SAXS results were validated through complementary transmission electron microscopy and atom probe tomography experiments, showing excellent agreement in both size and phase fraction. The presented methodology allows the successful capture of early-stage precipitation and provides an adaptable solution to background modelling challenges in non-isothermal SAXS experiments. This approach expands the applicability of SAXS for precipitation studies in structurally complex alloy systems.

## Abstract 2

A novel small-angle X-ray scattering modelling approach is introduced for the analysis of precipitation behaviour in metallic systems exhibiting scattering signals with streaks originating from the embedding matrix. This approach enables *in situ* tracking of the microstructural evolution, such as the volume fraction and size of second phases, during continuous heating, and it is showcased for a near-α Ti alloy.

## 1. Introduction

Small-angle X-ray scattering (SAXS) is a powerful technique to study structures on the nanometre scale and is widely used across fields ranging from soft matter and biology to materials science (Feigin & Svergun, 1987 ▸; Erdely *et al.*, 2019 ▸; Hamley, 2021 ▸; Jeffries *et al.*, 2021 ▸). Applied to the investigation of metallic systems where strengthening-relevant nanoscale precipitates form during controlled heat treatments, SAXS offers the unique capability to determine precipitate size distributions and volume fractions from a statistically significant sample volume (Fratzl, 2003 ▸; Okuda & Ochiai, 2013 ▸), thus providing a major advantage over localized techniques such as atom probe tomography (APT) or transmission electron microscopy (TEM).

Many studies have highlighted the effective application of SAXS to investigate precipitation mechanisms across various complex metallic systems, including Al-, Ni- and Ti-based alloys and steels (Graf *et al.*, 2022 ▸; Dumont *et al.*, 2005 ▸; Zhang *et al.*, 2016 ▸; Paris *et al.*, 1997 ▸; Andrews *et al.*, 2017 ▸; Tanaka *et al.*, 2020 ▸; Šmilauerová *et al.*, 2014 ▸). Precipitation hardening is of critical importance in alloy classes used for structural applications such as steels and Ti-based alloys, yet SAXS-related studies of these materials are comparatively rare. This is not only because the experiments typically require access to large-scale facilities (synchrotrons) but also due to the inherent complexity of data evaluation.

The complexity of SAXS data interpretation in metallic systems arises from several key challenges (Jeffries *et al.*, 2021 ▸). First, precipitates typically have complex morphologies and exhibit polydispersity, meaning they have a broad size distribution, requiring advanced modelling strategies. Second, multiple scattering effects, particularly in martensitic or lamellar matrices and often present in Ti-based alloys, introduce direction-dependent streaks in the 2D scattering patterns. In short, the microstructure in which the precipitates are embedded produces a scattering signal which makes direct interpretation and differentiation difficult. For instance, Schwaighofer *et al.* (2014 ▸) investigated the precipitation of carbides in an intermetallic TiAl alloy system using SAXS, where streaking in the SAXS patterns was observed and correlated using complementary microstructural characterization. The streaks found were attributed not only to the presence of a martensitic phase but also to ultra-fine γ-TiAl/α_2_-Ti_3_Al lamellar structures. In general, quantitative SAXS evaluation in metallic systems requires advanced fitting models, as the simple analytical approaches used for nanoparticles in dilute scatter-transparent solutions are often insufficient to give an accurate description of the morphology and distribution of precipitates. Zhang *et al.* (2016 ▸) highlighted the complexity involved in accurately modelling the scattering contributions in an Al alloy system. In their work, the authors emphasized the necessity of balancing the number of fitting parameters with a physically meaningful representation of the microstructure. In order to describe the scattering behaviour adequately across the full accessible range of the scattering vector **q**, they introduced a model comprising three distinct contributions, each supported by correlative TEM analysis. A recent study by Perrin *et al.* (2024 ▸) further emphasizes the ongoing effort to enhance the reliability and applicability of scattering techniques such as SAXS. The authors present methodological advances for analysing anisotropic precipitates in metallic systems, including the use of sample rotation to improve data interpretation. Their findings demonstrate the broader potential of SAXS across various alloy systems, highlighting the importance of continued methodological development in this field.

Over the past decade, De Geuser, Deschamps and co-workers (Deschamps & de Geuser, 2013 ▸; De Geuser *et al.*, 2012 ▸; De Geuser & Deschamps, 2012 ▸; Deschamps & De Geuser, 2011 ▸; De Geuser *et al.*, 2015 ▸; Deschamps & Hutchinson, 2021 ▸) have published several fundamental studies addressing these challenges, providing guidelines for SAXS-based precipitate analysis in metallic systems. Their work points out that computer-aided model fitting is a key tool for advanced SAXS analysis. Several studies have proposed practical strategies to address microstructural complexities, such as streaks in the scattering signal (Deschamps & de Geuser, 2013 ▸; De Geuser *et al.*, 2012 ▸; Feigin & Svergun, 1987 ▸). These methods typically involve either masking the streaked regions in the detector images or subtracting a constant background by selecting an initial reference signal. However, such approaches become ineffective in non-isothermal experiments, where the matrix continuously evolves due to simultaneous phase transformations and microstructural coarsening.

This study presents a novel SAXS modelling approach specifically developed to address the limitations associated with evolving matrices in non-isothermal experiments. The methodology was applied to a continuous-heating SAXS experiment performed *in situ* on a near-α Ti alloy. The proposed model enables a physically meaningful separation of matrix- and precipitate-related scattering contributions, allowing for accurate determination of precipitate size distributions and volume fractions throughout the heat treatment.

## 2. Experimental methods

### 2.1. Materials and heat treatment

The alloy investigated in this study is a near-α Ti alloy with a nominal composition of Ti–6Al–2Sn–4Zr–0.8Mo–3.6Nb–0.2Si–3Cu (wt%). Designed specifically for high-temperature applications, it includes Cu and Si to promote precipitation hardening via the formation of Ti_2_Cu and (Ti,Zr)_6_Si_3_ phases. A detailed microstructure characterization of this alloy, including the prevailing phases and its phase transformation behaviour during continuous heating, is described by Obersteiner *et al.* (2025 ▸).

**Figure 1:**
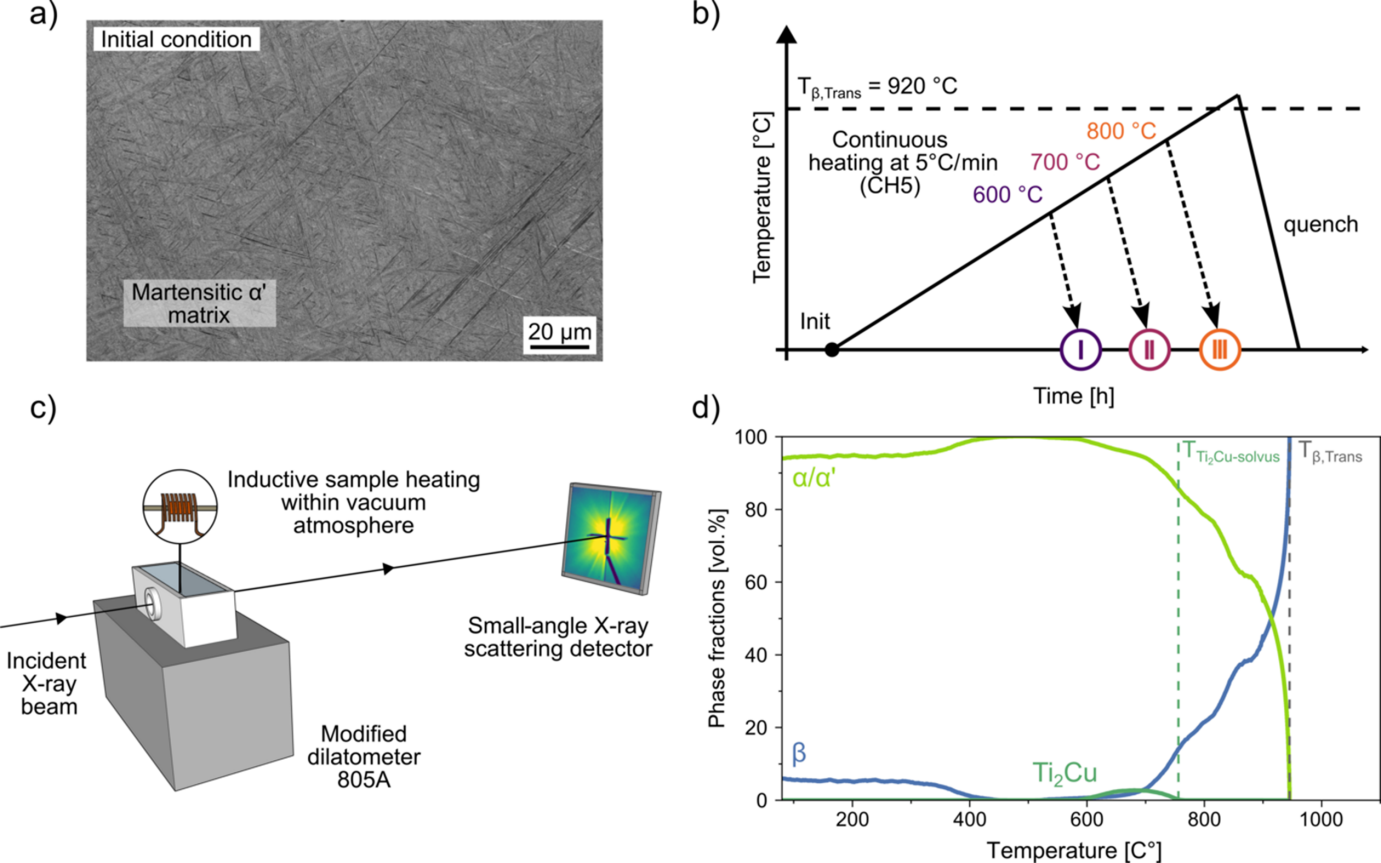
(*a*) Scanning electron microscopy–back-scattered electron (SEM-BSE) image of the initial sample condition, showing the fine martensitic α′ microstructure. (*b*) Thermal profile of the continuous heating experiment. The sample is heated at 5 °C min^−1^ from room temperature up to 900 °C, with subsequent rapid quenching. Selected states (I–III) were quenched from intermediate temperatures, *i.e.* 600, 700 and 800 °C, for microstructural analysis. (*c*) Schematic of the *in situ* SAXS setup on the P07 beamline at DESY. A modified DIL 805A dilatometer enables inductive heating under vacuum, while *in situ* SAXS measurements are performed in transmission geometry. (*d*) Evolution of phase fractions (α/α′, β and Ti_2_Cu) during CH5, derived from *in situ* high-energy X-ray diffraction (HEXRD) measurements. α/α′ and β represent the matrix phases of the alloy.

The Ti alloy was synthesized using a Mini Arc Melter MAM-1 from Edmund Bühler, Germany, under an inert Ar atmosphere. An alloy button (∼20 g) was produced and remelted five times to ensure chemical homogeneity. The solidified button was machined into rectangular samples with dimensions of 10 × 5 × 5 mm, which served as the final geometry for the synchrotron measurements. A homogeneous initial state for the SAXS measurements was achieved by solution heat treatment (SHT) above the β-transus temperature (*T*_β,Trans_ = 920 °C), followed by rapid quenching at 150 °C s^−1^. The SHT ensures a supersaturated martensitic condition [Fig. 1 ▸(*a*)] in which neither Cu- nor Si-rich precipitates are present. The resulting condition served as the starting point ‘Init’ for the *in situ* continuous-heating experiment, as shown in Fig. 1 ▸(*b*). The heating was performed at 5 °C min^−1^, hereafter referred to as CH5, to enable *in situ* observation of the precipitation process with high temporal resolution. The sample was heated in a modified DIL 805 A/D dilatometer from TA Instruments, Germany, under a vacuum atmosphere. The modification of the device includes two X-ray transparent windows, allowing for simultaneous monitoring of the sample by SAXS in transmission geometry [Fig. 1 ▸(*c*)]. Complementary HEXRD measurements were performed in a similar way to determine the evolution of phase fractions along the CH5 treatment to support the interpretation of the SAXS results. Fig. 1 ▸(*d*) illustrates the phase fraction diagram for the CH5 experiment, including the relevant phase transformations for this study.

### 2.2. Synchrotron setup

The *in situ* SAXS experiment was conducted on the P07 beamline at PETRA III, DESY, in Hamburg, Germany. A monochromatic X-ray beam with an energy of 73.8 keV, corresponding to a wavelength of 0.1680 Å, and a beam cross section of 200 × 200 µm were used. To record the SAXS signal, a Pilatus 2M detector with a total pixel matrix of 1475 × 1679 pixels and a pixel size of 172 × 172 µm was positioned at 10.66 m from the sample. This distance was calibrated using a silver behenate standard material. The small beam size combined with a precisely aligned beam stop allow the resolution of a *q* range from 0.006 to 0.4 Å^−1^ and enable the observation of precipitates ranging in diameter from 2 to 100 nm in real space. For SAXS data processing, air images were subtracted from the raw intensity data as background correction. Subsequently, additional corrections accounting for sample transmission, thickness, incident beam intensity and solid angle were applied. Azimuthal integration of the 2D detector images was performed using the *pyFAI* Python library (Ashiotis *et al.*, 2015 ▸). Finally, the macroscopic scattering cross section, *i.e.* the absolute intensity scale, was calibrated using a glassy carbon standard (NIST SRM 3600), following the procedure described in the literature (Allen *et al.*, 2017 ▸). Further data processing was carried out using Python scripts, followed by model fitting using the *SasView* software (Doucet *et al.*, 2024 ▸).

For the *in situ* HEXRD measurements, a PerkinElmer XRD flat-panel detector with a total pixel matrix of 2048 × 2048 pixels and a pixel size of 200 × 200 µm was positioned at 1.34 m behind the sample, with detector calibration carried out using LaB_6_ as a reference material. A beam size of 1 × 1 mm was used to increase the irradiated volume and improve grain statistics. After azimuthal integration, quantitative phase analysis was performed via Rietveld refinement using the *Profex* software (Doebelin & Kleeberg, 2015 ▸).

### 2.3. Complementary microstructure characterization

In order to validate the SAXS model and accurately interpret the obtained scattering data, complementary microstructural analysis was performed. For this purpose, the CH5 experiment was replicated *ex situ* in the laboratory, with samples quenched from defined temperatures to capture intermediate stages of the precipitation reaction, as indicated in Fig. 1 ▸(*b*). TEM was used to evaluate the morphology of the precipitates and to estimate their size. Therefore, thin foils (∼100 nm thickness) were prepared from the quenched samples by mechanical grinding, followed by dimpling and electrolytic polishing. Bright-field scanning transmission electron microscopy (BF-STEM) images and energy-dispersiveX-ray spectroscopy (EDS) maps were acquired using a Talos F200X G2 microscope from ThermoFisher Scientific, Germany, operated at 200 kV.

APT was carried out to give an accurate determination of the local chemical compositions of both the precipitates and the surrounding matrix with near-atomic resolution. This information is essential for calculating the scattering contrast, which is a key parameter for a quantitative evaluation of the precipitate phase fractions from SAXS. The SAXS signal’s intensity is sensitive to the electron-density contrast between the precipitates and the matrix. To this end, 100 µm thick ribbons were sectioned from the bulk material using mechanical cutting and grinding. These ribbons were then processed into APT tips via a sequential milling protocol, following the methodology outlined by Farabi *et al.* (2024 ▸) and Rielli *et al.* (2022 ▸), performed with a ThermoFisher Helios G4 Xe PFIB system (Germany). Data collection was conducted using a CAMECA LEAP 4000X Si straight-flight-path atom probe (USA) operating in laser-assisted evaporation mode. Experimental parameters included a 0.5% target evaporation rate, 200 kHz pulse frequency and 50 pJ laser energy. Samples were analysed under ultra-high vacuum conditions (<3.7 × 10^−11^ torr) at a constant temperature of 50 K throughout the analysis. Three-dimensional reconstructions of the atomic positions and compositional analysis of precipitates were conducted with the *AP Suite* software (Version 6.3.0.90; https://publcif.iucr.org/journals/misc/refs.html). The secondary electron images of the APT tips were used to reconstruct the data. Distinct poles/planes were not apparent in these datasets, and SEM-guided tip-profile initialization was used rather than crystallographic calibration. The SEM images were collected considering the stage tilt and then scale-calibrated. Finally, using the profile tracers provided by the software, the radial evolution function was calculated and used in the reconstruction. The isovalues used to determine the Cu-rich and Si-rich precipitates were defined by the data processing procedure introduced by Theska *et al.* (2018 ▸).

## 3. SAXS fitting strategy and precipitate modelling

**Figure 2:**
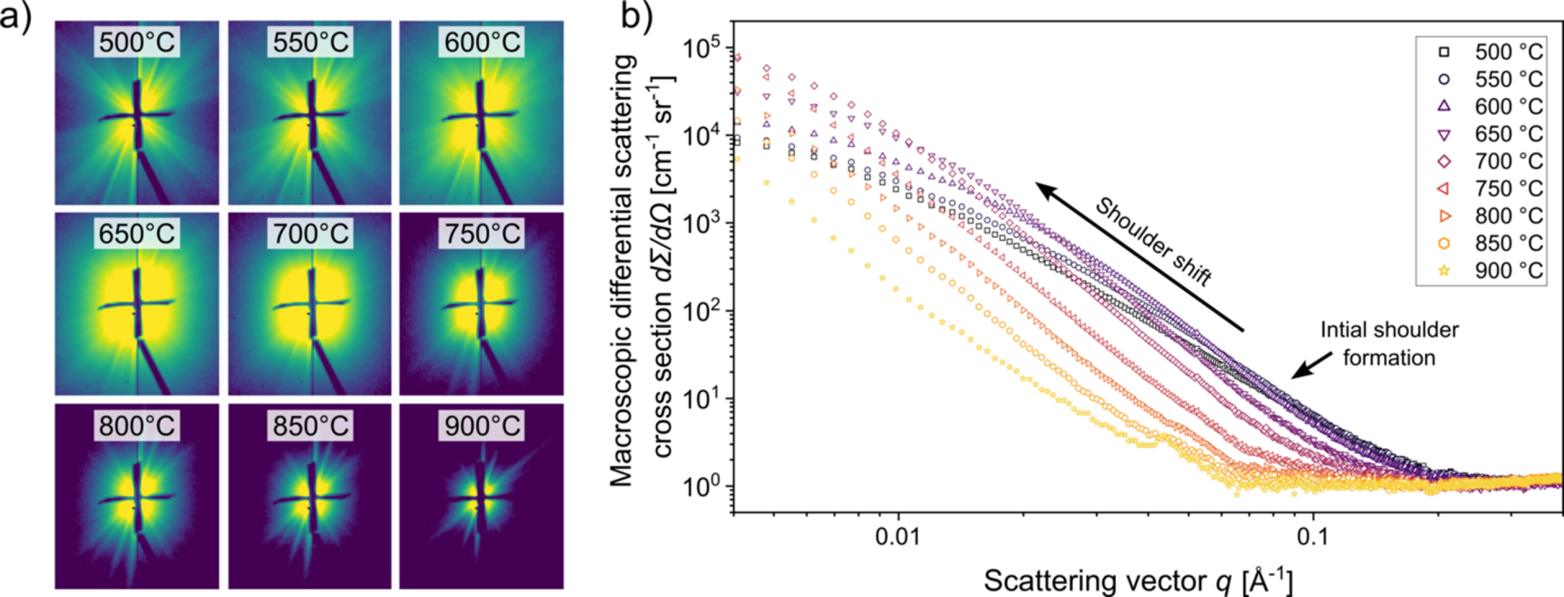
(*a*) Selected 2D SAXS detector images at different temperatures during continuous heating, showing the evolution of scattering intensity and streak features. (*b*) Azimuthally integrated SAXS intensity curves as a function of scattering vector magnitude *q* plotted on a double logarithmic scale, indicating the formation and shift of a shoulder at increased temperatures due to the nucleation and growth of precipitates, respectively.

Fig. 2 ▸(*a*) shows representative raw data in the form of 2D detector images at different temperatures throughout the CH5 treatment. Already at 500 °C, *i.e.* far below the expected onset temperature for precipitation, intense streaks are observed. This background signal, originating from the martensitic matrix, is already present in the initial condition at room temperature and remains largely unchanged until precipitation begins. In the following, the term ‘matrix’ refers to the microstructural background in which the precipitates are embedded. Initially, this matrix consists predominantly of α′ martensite. During the CH5 treatment, it gradually evolves into a mixed α/α′ structure and later, at higher temperatures, into a two-phase α + β microstructure. At 600 °C, a clear change in the scattering pattern emerges, marking the onset of precipitate nucleation and growth. In contrast to the streaked matrix signal, the new scattering contribution appears uniform, *i.e.* it is homogeneously distributed across the entire azimuthal direction, and it progressively increases in intensity, reaching a maximum at ∼650 °C. With further heating, the intensity of the signal diminishes again, indicating the dissolution of the previously formed precipitates. Complementary investigations using TEM and *in situ* HEXRD confirm that this decrease in scattering intensity is indeed associated with the dissolution of Ti_2_Cu precipitates, rather than continued growth beyond the SAXS detection limit. As *T*_β,Trans_ is approached, the streaked background signal weakens significantly, with only a few remaining streaks originating from the β grain structure persisting at 900 °C.

The handling of SAXS data in such complex metallic systems has been discussed in several studies, primarily by Deschamps and De Geuser (Deschamps & de Geuser, 2013 ▸; De Geuser *et al.*, 2012 ▸; De Geuser & Deschamps, 2012 ▸; Deschamps & De Geuser, 2011 ▸; De Geuser *et al.*, 2015 ▸; Deschamps & Hutchinson, 2021 ▸). When dealing with streaks that are not of main interest, two common approaches exist:

(i) Masking out the streaked regions. This is feasible when streaks are confined to a narrow angular range. However, masking results in data loss and reduced statistical accuracy. In the present case, Fig. 2 ▸(*a*) shows that the streaks extend over a wide azimuthal range, making masking impractical as it would eliminate most of the SAXS data.

(ii) Background subtraction of the initial SAXS signal. This method is commonly applied in isothermal experiments, where the background intensity is assumed to remain constant. However, in a continuous heating experiment such as CH5, the background intensity can change with increasing temperature and overlap with the precipitation reactions (De Geuser *et al.*, 2015 ▸). This prevents the use of a simple background subtraction approach for our study.

Nevertheless, azimuthal integration over the full 360° detector range, as commonly used in conventional background subtraction approaches, remains a valid initial step in our methodology. Although the initial SAXS patterns exhibit streaked features due to the preferred orientation of martensitic α′ laths [Fig. 2 ▸(*a*)], the present study does not aim to analyse or track these anisotropic features individually. Instead, the focus lies in modelling the evolution of the matrix background signal in a physically meaningful way throughout the CH5 experiment. In this context, averaging the SAXS intensity over the entire azimuthal range remains appropriate, as it enables a robust separation of background and precipitate contributions, even in the presence of directional features.

Thus, for a quantitative evaluation, the 2D SAXS images were azimuthally integrated over 360° to obtain 1D scattering profiles and plotted as a double-logarithmic diagram [Fig. 2 ▸(*b*)]. Several conclusions can be drawn from the resulting plots:

(i) The overall scattering intensity increases due to precipitation up to ∼630 °C.

(ii) A shoulder associated with newly formed precipitates emerges at around *q* ≃ 0.080 Å^−1^.

(iii) A systematic shift of the shoulder toward smaller *q* values with increasing temperature indicates growth of the precipitates.

With increasing temperature, the shoulder in the scattering profile progressively shifts toward smaller *q* values. After a certain temperature is reached, the shoulder moves beyond the accessible *q* range of the applied experimental setup, making further interpretation unreliable. In this regime, a reduction in scattering intensity can result from continued coarsening of the particles, from dissolution or from a combination of the two. Since these effects influence the scattering signal in a similar way, they cannot be unambiguously distinguished.

Following the azimuthal integration of the 2D detector images to 1D scattering curves [Fig. 2 ▸(*b*)], the integrated scattering intensity *Q* was calculated to track the evolution of the total scattering power with temperature, as it reflects the quantity of scattering elements. *Q* is defined as the total scattering intensity integrated over the entire scattering vector range (0 ≤ *q* ≤ ∞) after the raw intensity has been additionally corrected for background noise, known as Laue scattering, which fluctuated by only ∼5% throughout the experiment. The mathematical expression of *Q* is
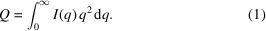
However, since SAXS experiments are constrained to a measurable range of *q*_min_ ≤ *q* ≤ *q*_max_, two assumptions are necessary to approximate the full integral:

(i) For *q* < *q*_min_, a triangular approximation was used, based on the lowest measured intensity and corresponding *q* value.

(ii) For *q* > *q*_max_, a Porod law extrapolation was applied to describe the asymptotic decay of the scattering signal at high *q*.

**Figure 3:**
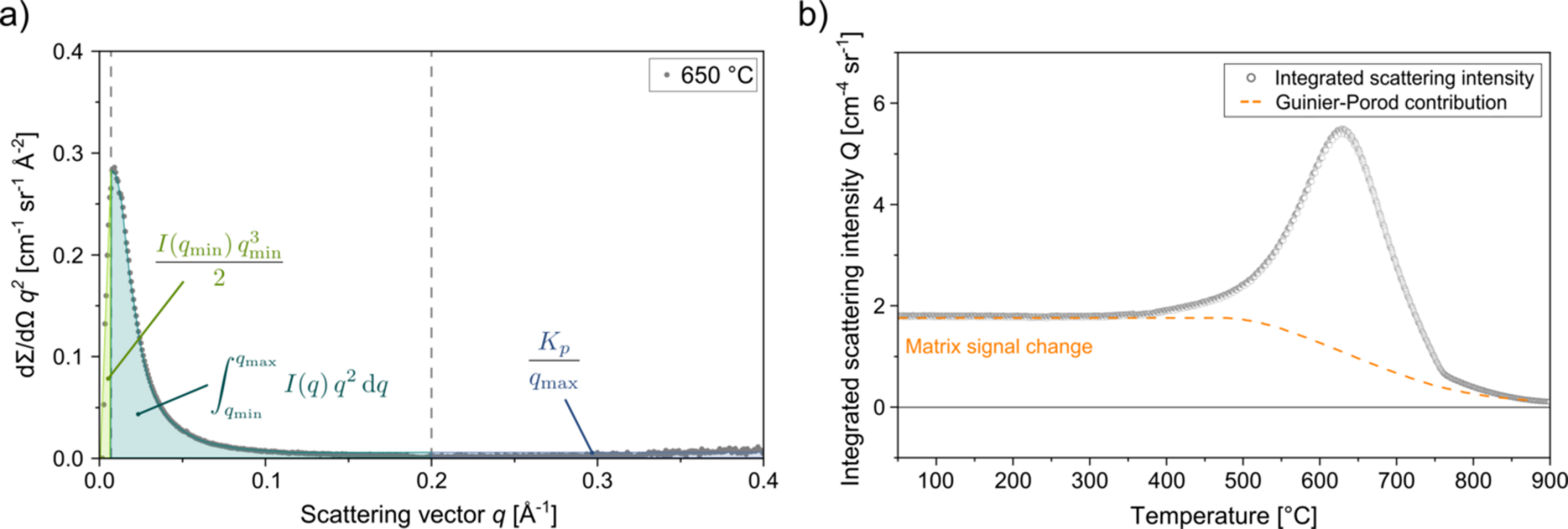
(*a*) Schematic representation of the integral approximation used to calculate the integrated scattering intensity *Q*. The total intensity is estimated by numerical integration between *q*_min_ and *q*_max_, with extrapolations for the low- and high-*q* regions using a triangular approximation and a Porod law extrapolation, respectively. (*b*) Evolution of the integrated scattering intensity *Q* over temperature. The circle symbols represent the total signal, while the dashed orange line corresponds to the Guinier–Porod background contribution, reflecting matrix signal changes.

Consequently, the numerical integration is only applied between *q*_min_ and *q*_max_. In summary, these assumptions can be expressed by the following equation:
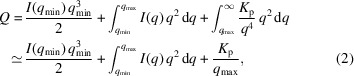
where *q* is the magnitude of the scattering vector and *I*(*q*) is the corresponding scattering intensity. The first and third terms represent approximations for the unmeasured low-*q* and high-*q* regions, respectively, while the middle term is evaluated numerically over the experimentally accessible range. *K*_p_ denotes the Porod constant, obtained by fitting the high-*q* region to a Porod law. A visual representation of this approach, showing the individual assumptions, is illustrated in Fig. 3 ▸(*a*). This formulation provides an effective estimate of the total scattering intensity *Q*. A detailed description of this approach is given by De Geuser & Deschamps (2012 ▸).

The temperature dependence of *Q* is plotted in Fig. 3 ▸(*b*). Up to approximately 400 °C, the integrated SAXS intensity remains constant, indicating that no significant structural changes or precipitation events occur in this range due to lack of thermal activation. Therefore, it can be assumed that the constant intensity level originates entirely from the matrix. Beyond this point, a pronounced increase in the integrated intensity *Q* is observed, reaching a maximum at around 630 °C. The curve profile, exhibiting a Gaussian-like shape, corresponds to the precipitation and growth of nanometre-sized particles. Precipitate dissolution is observed starting at about 760 °C, since the overall scattering intensity approaches zero. Note that the left and right flanks of the intensity peak terminate at different levels, highlighting the fact that changes in matrix scattering contribute to the overall SAXS signal. The contribution of the evolving background signal, illustrated by the orange dashed line, is approximated in this work using a Guinier–Porod function, the details of which are described in the following Section 3.1.

### 3.1. Guinier–Porod background

A Guinier–Porod function was used to model the evolving background signal of the martensitic matrix in the SAXS curves. This approach was effectively used in prior studies to describe the scattering behaviour of complex metallic matrices, particularly those with martensitic microstructures. For instance, Simm *et al.* (2017 ▸) applied a similar method in a small-angle neutron scattering (SANS) analysis of precipitation in maraging steels. The authors assumed this contribution to be constant under isothermal conditions. In the present case of continuous heating, however, both phase transformations and microstructural coarsening occur simultaneously, leading to a non-constant matrix signal.

**Figure 4:**
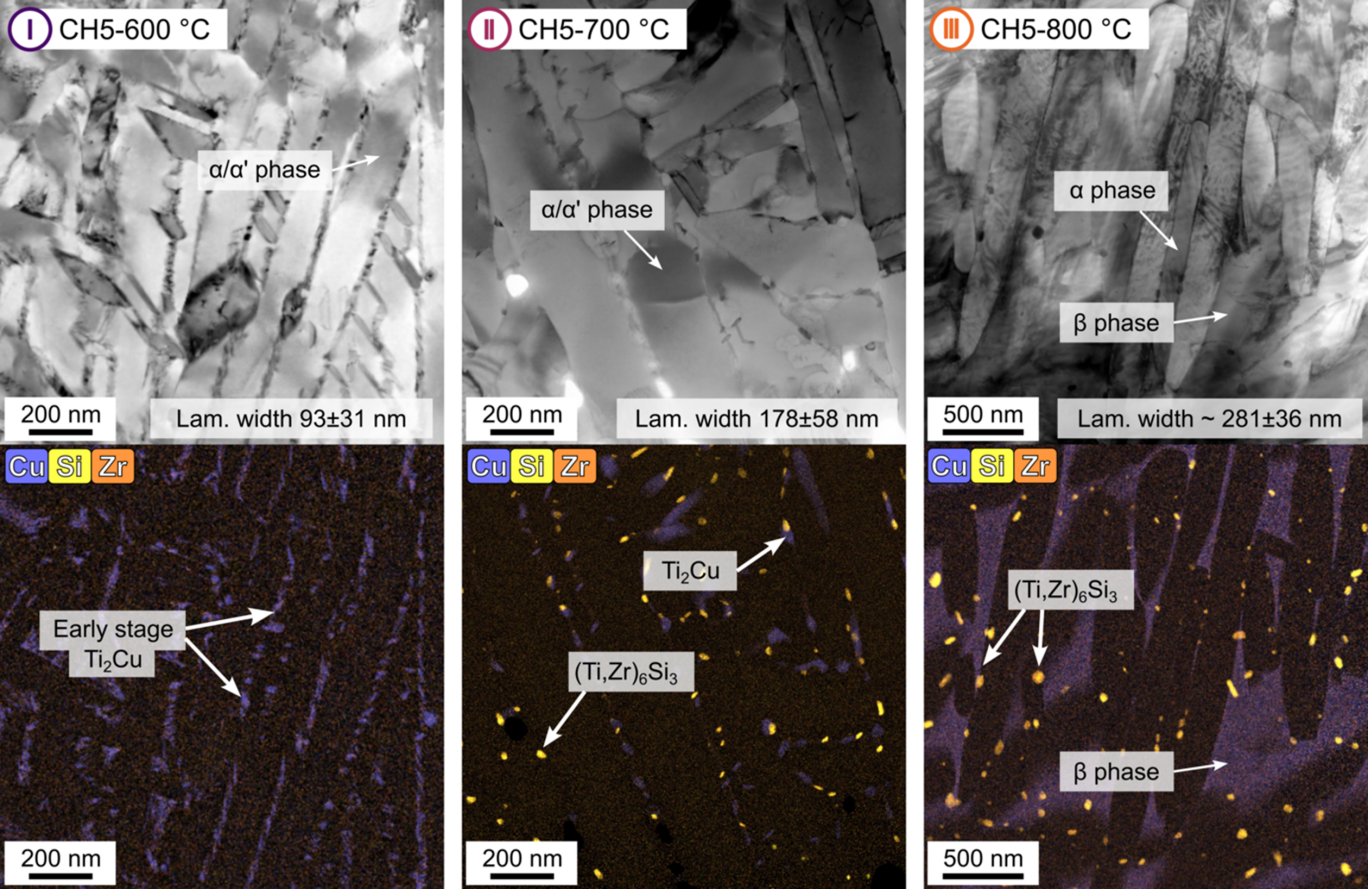
BF-STEM images (top row) and corresponding EDS elemental maps (bottom row) of Cu, Si and Zr for samples quenched from selected temperatures during the CH5 experiment: (I) 600 °C, (II) 700 °C and (III) 800 °C. The micrographs reveal the evolution of the lamellar α′/α matrix and the formation of Cu- and Si-rich precipitates.

In order to understand and quantify this evolving matrix signal better, complementary TEM analysis was performed on laboratory samples quenched from selected CH5 temperatures. Although quenching from elevated temperatures may induce additional martensitic phase transformations, this effect is considered small in the present study; the β phase fractions are relatively low at 600 and 700 °C, with values of less than 1 and 3 vol.% [Fig. 1 ▸(*d*)], respectively, where the Ti_2_Cu precipitation process mainly takes place. These *ex situ* replicas capture intermediate microstructural stages and were investigated using BF-STEM and EDS mapping. Fig. 4 ▸ summarizes the findings from the CH5-600 °C, CH5-700 °C and CH5-800 °C conditions. Additional SEM images of the *ex situ* replicas, providing further insight into the evolving microstructure at lower magnification, are presented in the supporting information (Fig. S1).

The top row in Fig. 4 ▸ displays BF-STEM micrographs, revealing the lamellar morphology of the α/α′ matrix. In Ti alloys, the α′ phase refers to a supersaturated martensitic variant of the hexagonal close-packed α phase, which forms upon rapid quenching and gradually transforms into equilibrium α and body-centred cubic β phases upon heating. In the CH5-600 °C condition, a fine lamellar structure is observed, with a measured lamellar width of λ ≃ 93 nm. This value, as well as those corresponding to higher temperatures, was derived from manual measurements of ∼50–100 lamellae per condition, and thus they should be considered as representative estimates based on the limited field of view. As the temperature increases, the interfaces between the lamellae become more defined, reflecting the gradual transformation of α′ into α + β. Despite this transformation, α and α′ share the same hexagonal crystal structure and, more importantly, exhibit very similar chemical compositions in the relevant temperature range. This allows for equivalent handling in the context of SAXS modelling. This simplification is further justified later in this paper, where it is shown that the difference in electron-density contrast remains below 3%. In the CH5-700 °C and CH5-800 °C conditions, coarsening of the lamellar structure is evident, with average widths increasing from ∼93 to ∼178 and ∼319 nm, respectively. The corresponding EDS maps (bottom row of Fig. 4 ▸) show the precipitation sequence. In CH5-600 °C, early-stage Cu-rich precipitates form between the lamellae, consistent with the onset of Ti_2_Cu precipitation [Fig. 1 ▸(*d*)]. The microstructure at CH5-700 °C shows additional formation of (Ti,Zr)_6_Si_3_ silicides besides coarsening of the Ti_2_Cu precipitates.

These findings from TEM micrographs already provide a clear understanding of the microstructural development, particularly the progressive coarsening of the lamellar structure during heating. To complement this observation with a focus on phase transformations, additional insights are gained from the HEXRD results [Fig. 1 ▸(*d*)]. They show that the β phase fraction is low at 600 °C (<1 vol.%) and 700 °C (<3 vol.%) and begins to increase significantly only at higher temperatures. Even at 800 °C, the β phase fraction reaches only ∼26 vol.%. This trend can also be seen in the TEM micrographs, particularly in the distribution of Cu. In CH5-600 °C and CH5-700 °C, Cu is only present in Ti_2_Cu precipitates between α/α′ lamellae. In contrast, in CH5-800 °C, Cu is fully dissolved in the β phase, which is now clearly noticeable as a distinct microstructural constituent. Additional elemental maps of other β-stabilizing elements such as Nb and Mo, provided in Fig. S2, further confirm this evolution of the β phase fraction with increasing temperature.

Overall, these results indicate that phase transformations do occur but remain limited throughout the main precipitation window (470 to 760 °C), which coincides with the temperature range in which the most pronounced changes in SAXS background intensity are observed [Fig. 3 ▸(*b*)]. In contrast, a pronounced coarsening of the lamellar structure occurs in this range, with lamellar widths increasing from ∼93 nm at 600 °C to ∼178 nm at 700 °C.

These observations support the assumption that coarsening of the lamellar structure is the main contribution to the background intensity evolution. Lamellar growth leads to a reducing number of scattering interfaces, and thus the matrix contribution to the overall intensity progressively decreases.

Consequently, the Guinier–Porod function (Hammouda, 2010 ▸), which is commonly used to describe scattering from hierarchical structures, was applied to estimate the contribution of the matrix. It is defined by
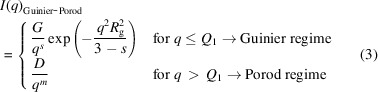
and
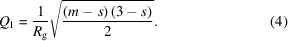
The radius of gyration *R*_g_ in this model provides an effective measure of microstructural feature size, such as lamellar thickness. The Guinier scale factor *G* defines the overall intensity of the scattering signal, *m* is the Porod exponent and *s* is an empirical value. *s* and *m* are used to describe the scattering behaviour of non-spherical objects in the Guinier regime or the Porod regime, respectively. In the case of lamellar/rod-like structures, *s* = 1.4 and *m* = 3 were used to reflect the martensitic substructure and the presence of diffuse internal interfaces (Hammouda, 2010 ▸). The intermediate value of *s* = 1.4 provides a reasonable approximation of the lath-like α′ morphology and was empirically found to yield the best fit to the initial SAXS profiles (from room temperature to 400 °C). To ensure a continuous transition between the Guinier and Porod regimes, the Porod scale *D* is computed from the Guinier equation evaluated at *Q*_1_. This guarantees a physically meaningful description of the scattering profile across the entire *q* range.

To describe the evolution of *R*_g_ with temperature, a classic parabolic grain growth law (Burke & Turnbull, 1952 ▸; Hillert, 1965 ▸; Cline, 1971 ▸) was applied. This is expressed as
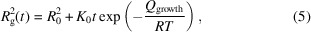
where *R*_0_ represents the characteristic lamellar size at time *t* = 0 s, *R* is the gas constant and *K*_0_ is a pre-exponential factor which depends on the alloy composition but is independent of the grain size.

In the present case, *R*_0_ was obtained by fitting the Guinier–Porod function to early-stage SAXS profiles (from room tem­perature to 400 °C) where the background signal remained constant. Subsequently, *K*_0_ was determined with equation (5) as a fitting function using this *R*_0_ and lamellar widths derived from TEM analysis at 600, 700 and 800 °C. This yielded a value of *K*_0_ = 1.78 × 10^−13^ m^2^ s^−1^. The growth behaviour follows an Arrhenius-type relationship, where an activation energy for grain coarsening of *Q*_growth_  = 100 kJ mol^−1^ was used. This value should be understood as an empirical fitting parameter within the simplified growth model rather than a physical constant of the specific alloy. A more detailed discussion and analysis are provided in the supporting information. These fitted parameters were then used to model the matrix signal dynamically throughout the heat treatment.

**Figure 5:**
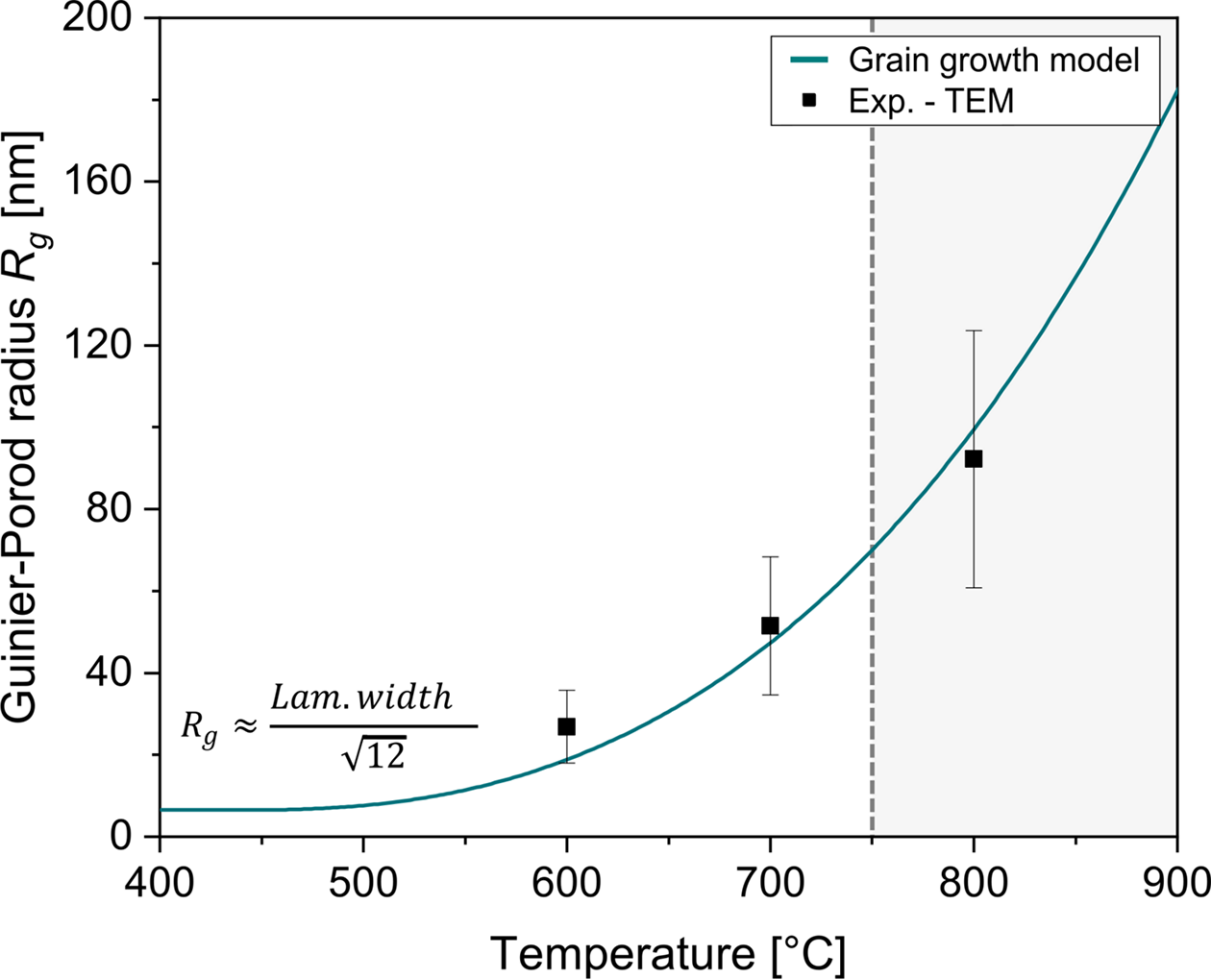
Temperature-dependent evolution of the Guinier–Porod radius *R*_g_ derived from a parabolic grain growth model. The experimental data points (black squares) were estimated from lamellar widths measured via TEM using the approximation 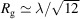. For each condition, ∼50–100 individual lamellae were evaluated to determine representative mean widths. The fitted curve represents the applied coarsening model used to describe the evolving background scattering in the Guinier–Porod function. The grey-shaded region indicates the temperature from which the precipitation reaction is almost completed [Fig. 1(*d*)] and the α → β transformation becomes increasingly pronounced, leading to significant changes in the matrix characteristics.

In this work, the model was initially parameterized using lamellar widths estimated from the TEM images, which led to dynamic adjustment of the background contribution in the SAXS fitting routine. Fig. 5 ▸ reflects the development of *R*_g_ with temperature in comparison with the experimental values from TEM. The lamellar widths were experimentally determined by manual measurements on individual lamellae in BF-STEM images. Due to the inherent limitations of 2D projections and the restricted number of measurable features, the resulting statistics are limited, leading to a relatively high standard deviation. However, this relatively simple coarsening model effectively captures the matrix evolution and allows for the robust separation of precipitate and matrix contributions during *in situ* SAXS evaluation.

## 4. Precipitation model

Following the implementation of the Guinier–Porod background to account for the matrix contribution, the next step in the SAXS evaluation involves selecting a suitable model to describe the precipitates.

For this purpose, it is essential to incorporate complementary characterization data, especially from TEM and APT, in order to constrain the model and reduce the number of free parameters to a physically meaningful range. As established earlier, TEM analysis revealed the presence of both Ti_2_Cu and (Ti,Zr)_6_Si_3_ precipitates during the CH5 experiment. Therefore, the potential simultaneous contribution of both phases to the SAXS signal requires careful consideration. Table 1 ▸ summarizes the individual chemical compositions and calculated scattering length densities (SLDs) for all phases present at relevant temperature conditions. The SLDs were determined by considering both the chemical composition and the corresponding atomic density of each phase. These values were then used to derive the scattering contrast (Δρ), which is defined as the difference in SLD between the precipitate and the surrounding matrix. Two important conclusions can be drawn on the basis of the complementary findings from TEM and APT:

(i) The Ti_2_Cu phase exhibits a scattering contrast about four times higher than that of (Ti,Zr)_6_Si_3_.

(ii) The volume fraction of silicides is significantly lower (by a factor of 10) according to estimates from TEM and further confirmed by prior HEXRD analysis as reported by Obersteiner *et al.* (2025 ▸).

These combined observations strongly suggest that the overall SAXS signal is dominated by the Ti_2_Cu precipitates. Consequently, a single-particle distribution model focused on Ti_2_Cu was applied, where the silicide contribution was neglected. To maintain a consistent input for SAXS modelling while avoiding the added complexity of changing Δρ with temperature, the scattering contrast between Ti_2_Cu and the matrix was approximated using the average of the calculated values at 600 and 700 °C. The deviation from the individual scattering contrasts relative to Δρ_mean_ = 7.25 × 10^−6^ Å^−2^ remains below 3%, justifying this simplification. This approach offers simplification of the fitting process while retaining sensitivity to the dominant scattering features of the system.

In order to describe the precipitate morphology of Ti_2_Cu accurately, an ellipsoidal model function (Feigin & Svergun, 1987 ▸) defined in the *SasView* software was used. The model accounts for the anisotropic shape observed in TEM and mathematically describes the scattering intensity *I*_ellipsoid_(*q*) for a single fixed orientation of the ellipsoid as
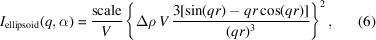
with the orientation-dependent effective radius defined by
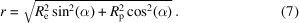
Here, *V* is the precipitate volume, Δρ is the SLD difference between the precipitate and the matrix, and *R*_p_ and *R*_e_ are the polar and equatorial radii of the ellipsoid, respectively. To reflect the realistic case of precipitates with random orientations, as expected in polycrystalline metallic alloys, the scattering intensity was averaged over all possible orientations. This was achieved by integrating over the full range of orientation angles α, resulting in
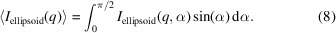
This orientation averaging ensures a statistically meaningful response and is more representative of the actual Ti_2_Cu precipitate morphology than a simplified spherical model. In order to represent realistic microstructural variation, a constant lognormal size distribution was applied to account for natural size variations within the precipitate population, further improving the accuracy of the SAXS evaluation (Staron *et al.*, 2002 ▸).

**Figure 6:**
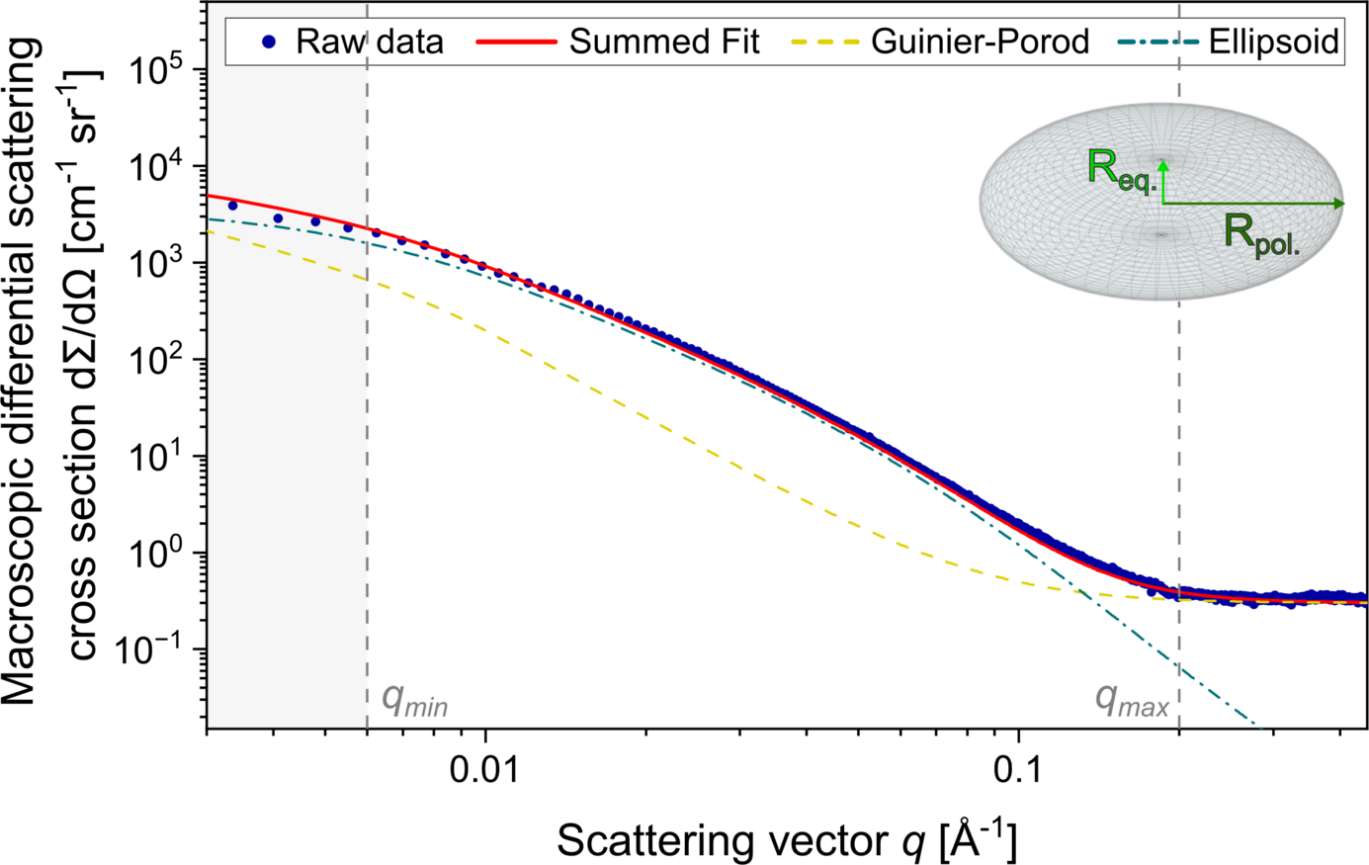
SAXS model fit at 550 °C, showing the combined contribution of matrix and precipitate scattering. The experimental data (blue dots) are fitted using the combined model (red line), which includes the Guinier–Porod background (yellow dashed line) to account for the matrix scattering, and the ellipsoidal model (blue dashed–dotted line) representing the Ti_2_Cu precipitates.

A combined SAXS model was developed to capture accurately the total scattering response during continuous heating. Therefore, contributions from the matrix background and the precipitate phase were summed. The matrix evolution is modelled using the Guinier–Porod function (Section 3.1), while the precipitate contribution is represented by the ellipsoidal model discussed above. To account for residual baseline fluctuations based on instrumental noise, a constant background term was included in the fitting procedure. The overall scattering intensity is therefore defined as
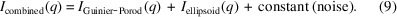
The effectiveness of this modelling approach is demonstrated in Fig. 6 ▸, which presents a representative SAXS fit at 550 °C. The excellent agreement between the experimental data and the model demonstrates that the applied methodology can robustly describe the experimental SAXS signal at this stage. However, to validate the model and its underlying assumptions, complementary methods are indispensable to confirm whether the approach enables a physically meaningful separation of matrix and precipitate intensity contributions and allows for an accurate determination of phase fractions and size evolution.

## 5. Model results and cross validation

**Figure 7:**
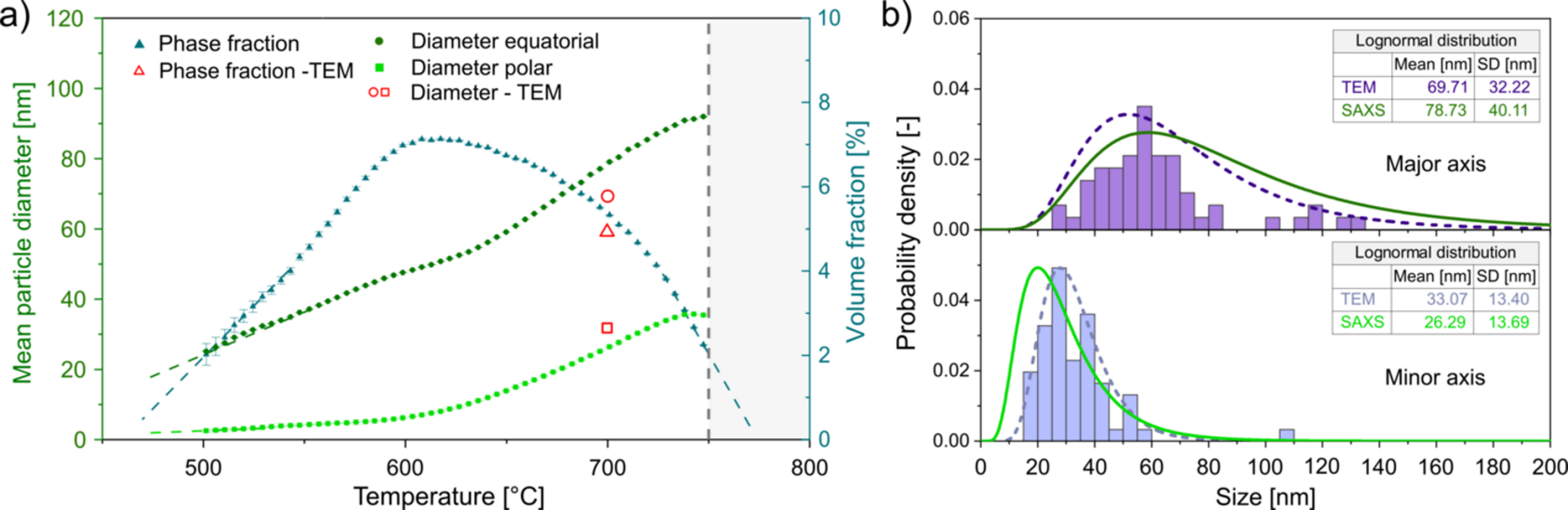
(*a*) Temperature-dependent evolution of the equatorial and polar diameters of Ti_2_Cu precipitates and evolution of volume fraction obtained from SAXS fitting, with error bars representing fitting uncertainties from *SasView*, and comparison with experimentally determined values for 700 °C using TEM. The shaded region indicates the fitting limit due to *q*-range constraints. (*b*) Lognormal size distributions of the major and minor precipitate axes, including the standard deviation SD for 700 °C, derived from SAXS and TEM.

The SAXS evaluation approach developed here enables a quantitative assessment of the size evolution and volume fraction of Ti_2_Cu precipitates during a continuous heating experiment. The corresponding results are summarized in Fig. 7 ▸.

Fig. 7 ▸(*a*) shows the temperature-dependent evolution of the precipitate diameters derived from the ellipsoidal model, as well as the evolution of the Ti_2_Cu phase fraction obtained from SAXS. According to the model, precipitation begins at approximately 470 °C, significantly earlier than is detectable by *in situ* HEXRD [Fig. 1 ▸(*d*)]. The initial values of the polar and equatorial diameters suggest the formation of thin highly elongated precipitates. This observation is consistent with the results of the TEM analysis (Fig. 4 ▸), which show Ti_2_Cu nucleating along α/α′ lamellar interfaces, an energetically favourable site for anisotropic growth. With increasing temperature, both diameters grow progressively, indicating continuous particle coarsening. The aspect ratio remains relatively stable, suggesting that the precipitate morphology is preserved throughout the heat treatment. These findings are further supported by the APT results, with corresponding atom maps provided in Figs. S3 and S4. Farabi *et al.* (2025 ▸) similarly reported that Cu-rich and Si-rich intermetallic precipitates nucleated predominantly along phase boundaries in a conjugated manner, exhibiting comparable morphologies to those observed in the present study.

In order to validate the model, precipitate sizes and phase fractions were additionally estimated from TEM-based image analysis for the CH5-700 °C condition, where the Ti_2_Cu precipitates exhibited a clearly distinguishable morphology. In this case, ∼50 individual precipitates were evaluated using threshold-based image analysis to determine both size and volume fraction. From these measurements, mean values for the major and minor axes were derived to enable a direct comparison with the SAXS results. For CH5-600 °C, no reliable TEM-based quantification could be performed. Although early-stage Cu-rich features are visible in the corresponding TEM-EDS maps, their diffuse and fringe-like appearance prevents consistent segmentation and size determination using image thresholding methods. However, as stated above, the thin and elongated morphology observed in TEM qualitatively supports both the SAXS results and the interpretation from the applied modelling approach. The experimentally derived precipitate sizes from the CH5-700 °C condition are overlaid in Fig. 7 ▸(*a*) and show an excellent agreement with the SAXS results. At higher temperatures (*T* > 740 °C), the fitted diameters approach the limits of the experimentally accessible *q* range, as marked in the diagram. This region should be interpreted cautiously due to increased uncertainty from extrapolation. The onset of precipitation at ∼470 °C, peak volume fraction at ∼620 °C and dissolution above 750 °C correspond well to the expected precipitation window. A cross check with the results from TEM-EDS-based image analysis at 700 °C yielded a Ti_2_Cu volume fraction of ∼4.8 vol.%, closely matching the SAXS result.

Fig. 7 ▸(*b*) provides a further comparison between SAXS and TEM results by plotting the lognormal size distributions of the major and minor axes at 700 °C. While slight differences are observed in the tails of the distributions, the mean sizes and polydispersity parameters are in good agreement, emphasizing the validity of the SAXS-based model in capturing both the shape and size distribution of the precipitates within a complex lamellar matrix. The comparative values from the TEM measurements are based on a limited field of view and thus offer a limited statistic, further highlighting the advantage of the SAXS method. Nevertheless, this strong agreement supports the robustness of the combined SAXS evaluation approach in resolving precipitate evolution during a dynamic thermal cycle.

## 6. Conclusion

This work presents a SAXS evaluation approach tailored for metallic systems with complex microstructures, addressing a key limitation in the analysis of streaked 2D scattering patterns – a recurring challenge in alloys such as Ti-based alloys and steels with fully or partly martensitic starting microstructures. By incorporating a Guinier–Porod background model linked to a simple grain coarsening law, this novel approach enables the description of a dynamically changing background signal, and thus a physically meaningful separation of matrix and precipitate scattering contributions, during continuous heating experiments.

Although the microstructural evolution in Ti-based alloys during heating can include multiple overlapping phenomena, such as α′ → α + β transformation, phase fraction changes and coarsening, this relatively simple modelling framework already yields highly consistent and well interpretable results. Cross-validation with complementary techniques, like TEM, EDS and APT, confirms that the SAXS-derived precipitate sizes and phase fractions are both accurate and representative of the actual microstructure.

While the model can be further refined in future work to account better for transformations and evolving phase fractions, the present study demonstrates that even a simple but physically meaningful background description can enable robust SAXS analysis in challenging alloy systems. Beyond the specific case of a near-α Ti alloy, this work highlights the broader potential of SAXS as a statistically powerful high-resolution tool for *in situ* studies of structural materials.

In the present study, a robust SAXS evaluation approach has been established to describe the evolving matrix scattering contribution during continuous heating, enabling the tracking of Ti_2_Cu precipitate nucleation, growth and dissolution from the earliest stages. Overall, the methodology provides a transferable framework for future SAXS investigations of precipitation in complex lamellar microstructures, with particular relevance for Ti-based alloys, steels and other complex engineering materials.

## Supplementary Material

Additional figures. DOI: 10.1107/S1600576725008489/jl5123sup1.pdf

APT-Tip-movie_CA-600C. DOI: 10.1107/S1600576725008489/jl5123sup2.mp4

APT-Tip-movie_CA-700C. DOI: 10.1107/S1600576725008489/jl5123sup3.mp4

## Figures and Tables

**Table 1:** Chemical compositions (in atomic percent) of the matrix and precipitate phases at selected temperatures during the CH5 treatment, as determined by APT Corresponding atom maps are provided in the supporting information, Figs. S3 and S4. The scattering length densities (SLDs) were calculated from the measured compositions and corresponding atomic densities.

		Chemical composition (at.%)								
Condition	Phase	Ti	Al	Sn	Zr	Mo	Nb	Si	Cu	SLD (×10^−6^ Å^−2^)
600 °C	α/α′ phase	82.97 ± 0.04	11.83 ± 0.03	0.72 ± 0.1	1.62 ± 0.01	0.06 ± 0.01	1.62 ± 0.01	0.47 ± 0.01	0.65 ± 0.01	34.66
	Ti_2_Cu	69.13 ± 0.05	2.22 ± 0.01	0.12 ± 0.01	2.82 ± 0.01	0.07 ± 0.01	0.66 ± 0.01	0.44 ± 0.01	24.49 ± 0.04	42.13
700 °C	α phase	82.9 ± 0.09	11.27 ± 0.07	0.7 ± 0.02	1.49 ± 0.03	0.05 ± 0.02	1.96 ± 0.03	0.38 ± 0.02	0.79 ± 0.02	35.02
	Ti_2_Cu	71.69 ± 0.08	2.71 ± 0.03	0.11 ± 0.01	2.57 ± 0.03	0.05 ± 0.01	0.81 ± 0.01	0.21 ± 0.01	22.39 ± 0.07	42.04
	(Ti,Zr)_6_Si_3_	34.82 ± 0.65	1.55 ± 0.17	1.88 ± 0.19	20.45 ± 0.54	0.41 ± 0.09	1.53 ± 0.21	38.57 ± 0.66	0.44 ± 0.11	36.67

## Data Availability

The data supporting the findings of this study are available from the corresponding author upon request.
