# Supplementary material for: Advancing small-angle X-ray scattering for complex metallic systems: Ti2Cu precipitation in a martensitic near-α Ti alloy
Source: J Appl Crystallogr. 2025 Nov 4;58(Pt 6):2006–17. doi: 10.1107/S1600576725008489 (PMC12810513; doi:10.1107/S1600576725008489)
Supplement: Supplementary file 1 [file j-58-02006-sup1.pdf]

JOURNAL OF
APPLIED
CRYSTALLOGRAPHY

Volume 58 (2025)

Supporting information for article:

Advancing SAXS for complex metallic systems: Ti_2Cu precipitation in a martensitic near-α Ti alloy

David Obersteiner, Sabine C. Bodner, Helmut Clemens, Andreas Landefeld, Ehsan Farabi, Sophie Primig, Peter Staron, José L. Neves, Thomas Klein and Michael Musi

Supplementary material

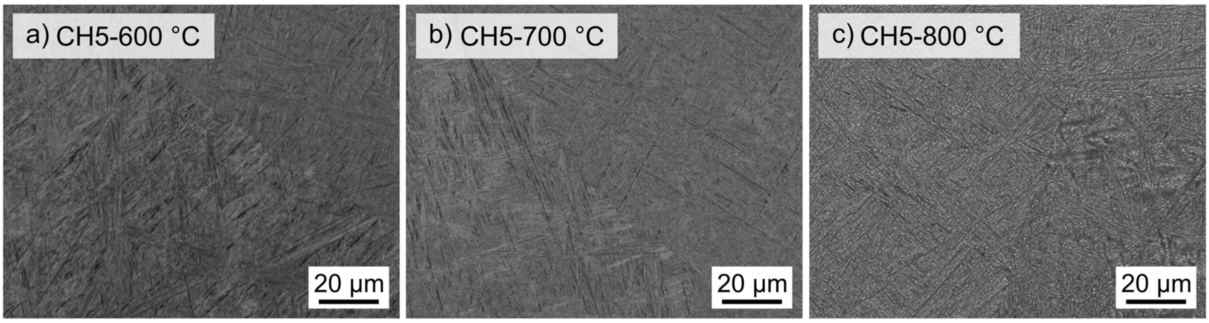

Figure S1. SEM images (BSE mode) of the ex situ replicas quenched from CH5 heat treatment at (a) 600 °C, (b) 700 °C, and (c) 800 °C, showing the evolving lamellar microstructure with increasing temperature.

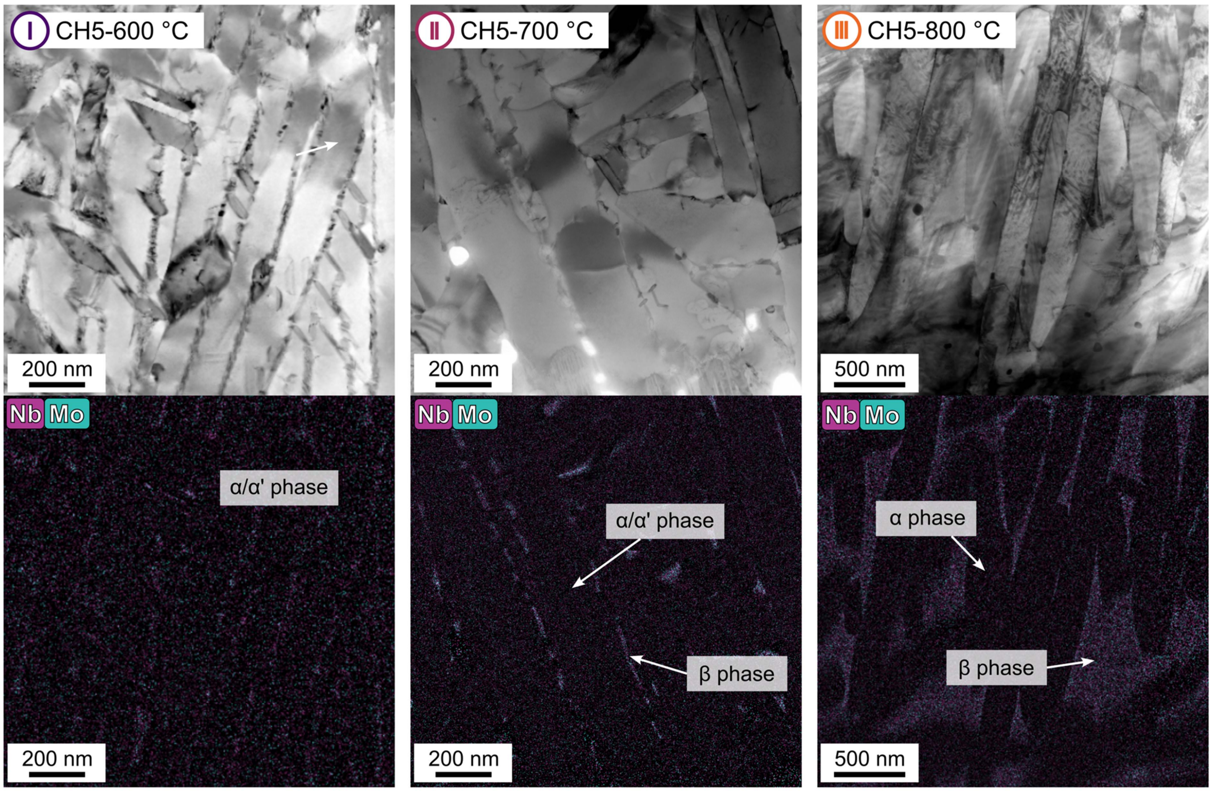

Figure S2. BF-STEM images (top row) and corresponding EDS elemental maps (bottom row) of Nb, and Mo for samples quenched from selected temperatures during the CH5 experiment: (I) 600 °C, (II) 700 °C, and (III) 800 °C. The micrographs reveal the evolution of β phase with increasing temperature.

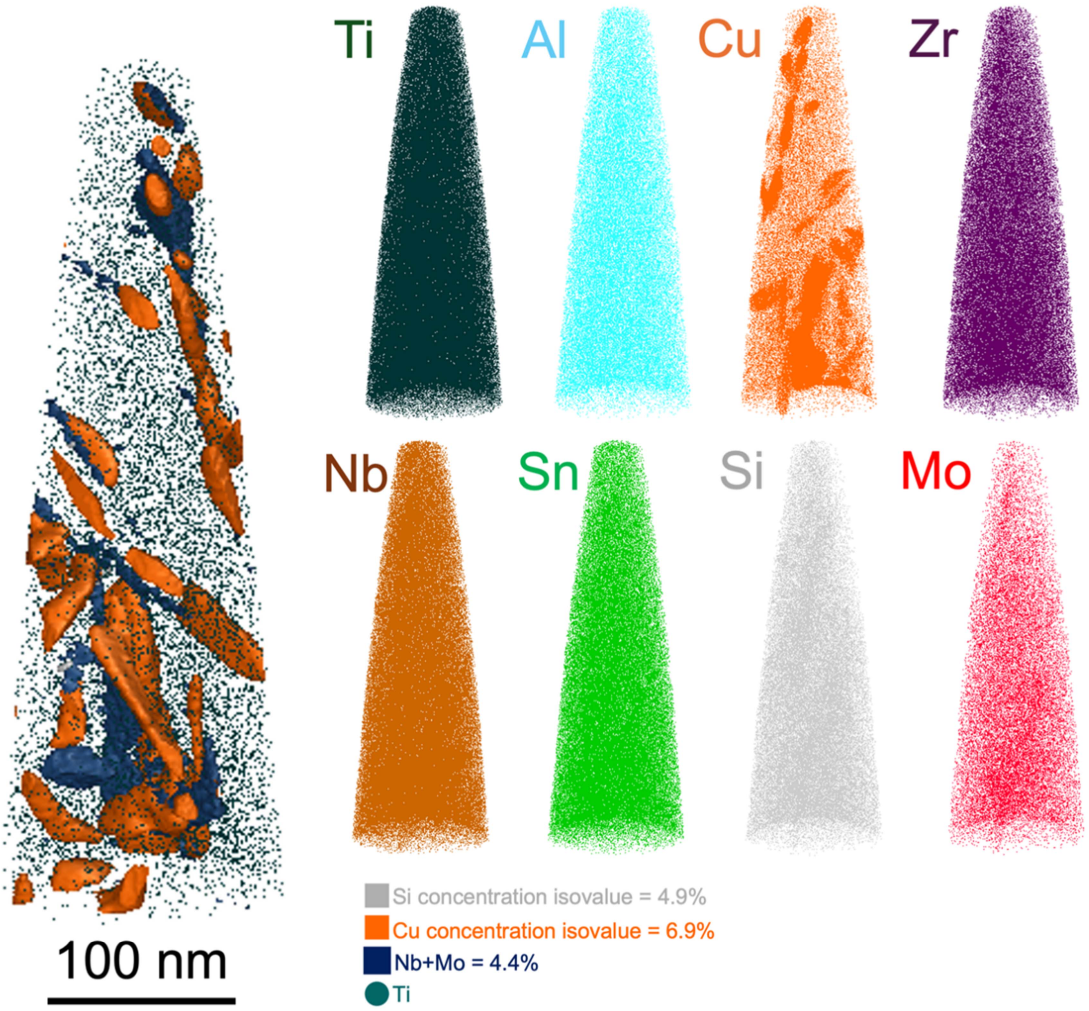

Figure S3. 3D reconstructed Ti atom maps of CH5-600 °C condition with Ti_2Cu, Si-rich precipitates, and β phase regions as identified by 6.9 at.% Cu, 4.9 at.% Si and 4.4 at.% Nb+Mo isosurfaces, respectively.

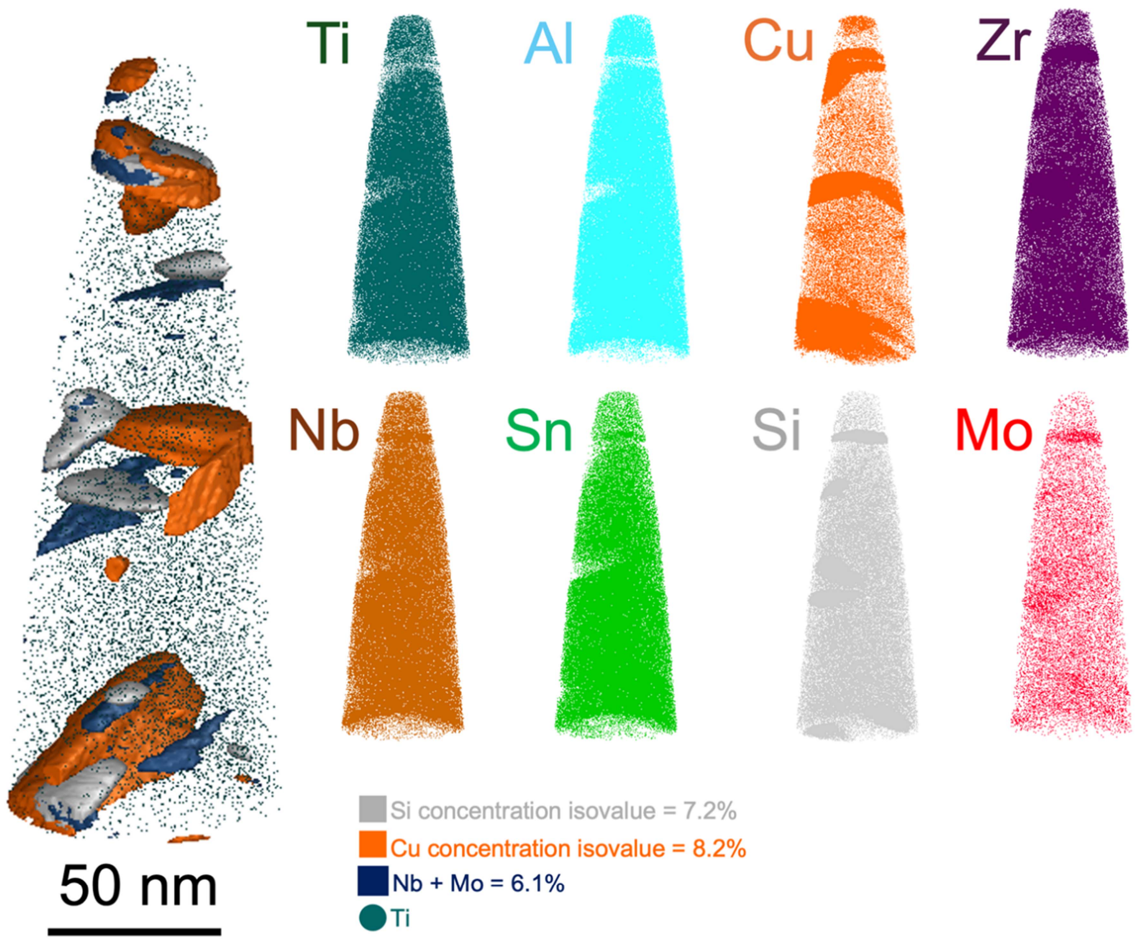

Figure S4. 3D reconstructed Ti atom maps of CH5-700 °C condition with Ti_2Cu, Si-rich precipitates, and β phase regions as identified by 8.2 at.% Cu, 7.2 at.% Si and 6.1 at.% Nb+Mo isosurfaces, respectively.

Grain coarsening model and choice of Q_{growth}

To describe the temperature-dependent evolution of the matrix scattering contribution, we employed a classical parabolic grain growth law, adapted to the lamellar structure by treating the radius of gyration R_g as a representative "characteristic size." This approach enabled a simplified but tractable modelling of the evolving matrix background in the SAXS data. Since no established coarsening models exist for complex lamellar dual-phase alloys like the one investigated here, assumptions were necessary. In this context, the activation energy for grain growth, Q_{growth}, was treated as an empirical fitting parameter rather than a material constant. To evaluate the sensitivity of the model, we tested a range of Q_{growth} values between 100 kJ/mol and 250 kJ/mol, re-optimizing the corresponding K_0 value in each case to match the experimental lamella width data at 600 °C, 700 °C, and 800 °C. The resulting fits are shown in Fig. S5. While higher values of Q_{growth} fall within the range typically

reported for Ti alloys, they systematically underestimate the coarsening observed experimentally. The selected value of Q_{growth} = 100kJ/mol provides the best overall match and is thus retained in the model. However, we emphasize that this value is model-dependent and does not represent a fundamental property of the alloy. Instead, it enables a consistent description of the background evolution in the SAXS fitting routine and is appropriate within the assumptions of the applied growth law.

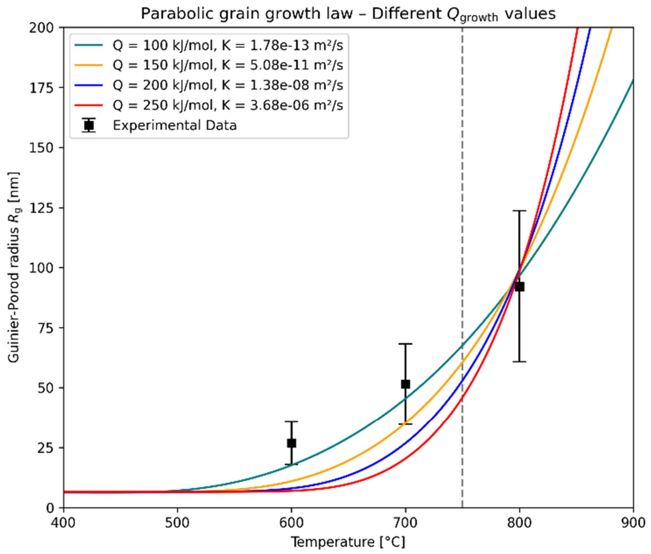

Figure S5. Sensitivity of the parabolic grain growth model to different values of the activation energy Q_{growth}. The comparison highlights that the simplified growth model best describes the observed coarsening behaviour when using Q_{growth} =100kJ/mol, even though this value should not be interpreted as a physical material constant.
